# Prevalence of diagnosis and direct treatment costs of back disorders in 644,773 children and youths in Germany

**DOI:** 10.1186/1471-2474-11-193

**Published:** 2010-08-28

**Authors:** Elke B Ochsmann, Carlos L Escobar Pinzón, Stephan Letzel, Thomas Kraus, Martina Michaelis, Eva Muenster

**Affiliations:** 1 Institute for Occupational and Social Medicine, Medical Faculty, RWTH Aachen University, Aachen, Germany; 2 Institute of Occupational, Social and Environmental Medicine, University Medical Center of the Johannes Gutenberg University Mainz, Mainz, Germany; 3 Research Centre for Occupational and Social Medicine (FFAS), Freiburg, Germany

## Abstract

**Background:**

Many authors have reported about the high prevalence rates of self-reported back pain in children. Nevertheless, little is known about the diagnosis of back disorders - regardless of whether the diagnosis is associated with back pain or not. Therefore, the aim of this study was to analyse the prevalence rates and costs of diagnosis of back disorders in childhood and youth.

**Methods:**

We conducted a secondary data analysis of a large, population based German data set (2,300,980 insurants of statutory health insurance funds) which allowed for identification of prevalence rates of diagnoses of back disorders in children (age group 0-14 years) and youths (age group 15-24 years) using three digit ICD-10 codes for dorsopathies (**M40 - M54: **kyphosis and lordosis; scoliosis; spinal osteochondrosis; other deforming dorsopathies; ankylosing spondylitis; other inflammatory spondylopathies; spondylosis; other spondylopathies; spondylopathies in diseases classified elsewhere; cervical disc disorders; other intervertebral disc disorders; other dorsopathies, not elsewhere classified; dorsalgia). Direct treatment costs were calculated based on the real incurred costs for cases with a singular diagnosis of a back disorder. Wherever possible, the results of the random sample were extrapolated to all insurants of statutory health insurance funds (i. e., about 90% of the German population).

**Results:**

We found prevalence rates for the diagnosis of back disorders to range between 0.01 - 12.5%. "Scoliosis" (M41) and "dorsalgia" (M54) were the most frequent diagnoses in both age groups. Based on these results, it was calculated that in 2002 alone, approximately 1.4 million children/youths in Germany were diagnosed with "dorsalgia" (M54), and that the direct costs for back disorders in childhood and youth accounted for at least 100 million Euros.

**Conclusions:**

Instead of focusing on the individual, and self-reported disorder or disability, this analysis allowed for the detailed evaluation of medical experts' opinion on back disorders in childhood and youth and for a more objective or public health oriented insight in the topic of diagnosis of back pain and other back disorders. However, due to the methodological limitations by using ICD-10 coding, standardized random validity checks of population based data sets should be mandatory.

## Background

Back pain is a cardinal symptom for various diseases of the back or spine, that is, for "dorsopathies" or back disorders. It is also acknowledged to be a common condition in childhood and youth [1-4], with self-reported one-year prevalence rates of up to 83% [1], and might - even at this early age - be associated with difficulties and restrictions in everyday life [5,6]. Although various authors report high prevalence rates of pain and especially back pain in children, adolescents, and youths in Germany [e. g. [7-9]] with three-month prevalence rates between 30.2 - 49.0%, all of these reports focus on self-reported back pain. And while self-reported complaints of back pain seem to be relatively common in these age groups, the number of children seeking medical care because of back pain seems to be rather unclear and might be influenced by age or regional aspects and furthermore it might have increased over the years. Olsen et al. [10] for example reported in 1992 that 7% of the then examined U.S. adolescents with back pain sought medical attention. In 1999, 23% of Belgian school children (aged nine) with low back pain had sought medical help from a doctor or a physiotherapist [11], and in 2005, it was reported that 32% of Tunesian children and adolescents required medical help because of back pain [12]. With regard to German children, Roth-Isigkeit et al. [8] reported that 53% of German children with back pain visited a doctor. These immense differences in self-reported health care utilization when experiencing back pain led to the conclusion, that besides the analysis of self-reports on back pain, it might be worthwhile to consider back pain from a more population based or public health point of view and evaluate prevalence rates and costs of diagnosis in children, youths and adolescents in existing data sets of health insurances. As, to our knowledge, little is known about the prevalence rates of diagnosed back disorders in general in childhood and/or youth, we extended our research approach, notwithstanding the possible association of these disorders with back pain. Therefore this article addresses the question of prevalence rates of diagnosis, as well as direct costs of back disorders in German children and youths. An extensive, population based dataset focussing on all persons insured in statutory health insurance funds (approximately 90% of the German population) was used to answer this question.

## Methods

### Data set

The analyzed data set originally consisted of all insurants of statutory health insurances in Germany in the year 2002. About 350 statutory health insurance funds, the 23 associations for statutory health insurance physicians, the German Federal Insurance Authority, the Federal Pensions Office for Salaried Employees as well as the German Institute of Medical Documentation and Information (DIMDI) contributed to this data set which comprises about 90% of the German population (i.e. approximately 72 million persons). Along with other factors, the data set surveys the insurants' medical diagnosis as three digit ICD-10 codes and costs incurred by in- and outpatient treatment. For scientific evaluation a 3% probability sample (birthday sample) was drawn out of the whole cohort and is currently available for scientific evaluations by the Federal Office of Statistics. Every insured or co-insured person, who was born on the 11^th ^of any month and was insured for at least one day in one of the statutory health insurance funds, was included in this birthday sample, comprising altogether 2,300,980 persons. The basic data file contains information about birth year and gender of the insurants, as well as the ICD-10 codes of all accounting cases. An analysis of costs per accounting case with regard to specific ICD-10 diagnosis was conducted by linking the basic data file with the data files regarding "outpatient treatment" and "inpatient treatment & operation" using the insurant's pseudonymous insurance number, which is included in all data files. Further details on the dataset and its collection methods can be gathered from the German brochure "Federal Office of Statistics and Länder Statistical Offices, Research Data Centers, Working Paper no. 22"[13]. Information about risk factors for diseases (e. g., psychosocial variables, body mass index, occupation, sports) was not included in this data set.

The gathering of data and the here presented analysis are in accordance with the German data protection act and in accordance with local legislation. All patients are aware of the possibility of anonymous evaluation of their insurance data. This research conforms to the Helsinki Declaration. Ethics review board approval was not obtained for these secondary analyses of an existing public access dataset.

### Back disorders

In this study, we focused on three-digit ICD-codes (M40-M54) in order to report on back disorders in children and youths http://www.who.int/classifications/icd/en. The different conditions summarized under these codes are depicted in table 1.

**Table 1:** back disorders according to ICD-10 (2002 version)

M40-43: deforming dorsopathies	M40: kyphosis and lordosis
	M41: scoliosis
	M42: spinal osteochondrosis
	M43: other deforming dorsopathies
M45-M49: spondylopathies	M45: ankylosing spondylitis
	M46: other inflammatory spondylopathies
	M47: spondylosis
	M48: other spondylopathies
	M49: spondylopathies in diseases classified elsewhere

M50-M54: other dorsopathies	M50: cervical disc disorders
	M51: other intervertebral disc disorders
	M53: other dorsopathies, not elsewhere classified
	M54: dorsalgia

### Age groups

The definition of children (0-14 years) and youths (15-24 years) was chosen in accordance with the definition of the United Nations General Assembly http://www.un.org. The a priori created age groups were analyzed. According to the chosen definition, altogether 15.6% of the collective have to be referred to as children, and 12.4% have to be referred to as youths.

### Statistical methods and calculation of costs

The above described data set was used for a secondary data analysis. For the evaluation, SAS^®^-programs were written and sent to the Federal Office of Statistics, where the actual analysis was carried out in two steps. First, the number of the insured children and youths with a relevant diagnosis, as well as the prevalence rates of dorsopathies in the 3% birthday sample (three-digit ICD-10 code, ICD-10 SGB V1.3 (2000-2003)) were calculated. All persons with a relevant diagnosis/ICD-code (singular diagnosis and/or multiple diagnoses) were included in the calculation of prevalence rates. These results were then used to extrapolate the number of affected children and youths insured with statutory health insurers in 2002, multiplying them by 33.3. Second, the treatment costs, which were based on fee items according to the German EBM of the year 2002 (EBM: **E**inheitlicher **B**ewertungs**m**aßstab für ärztliche Leistungen, common valuation standard for medical treatment), were calculated in two steps. In the first step, we only regarded cases (inpatient and/or outpatient cases) where the relevant musculoskeletal diagnosis was found to be the only diagnosis (singular diagnosis), leaving out cases where the relevant disease was coded as one of more diagnoses, as the existence of other diagnoses (other musculoskeletal diagnosis or other than musculoskeletal diagnosis) might have influenced the corresponding costs. As having a singular diagnosis was only true for a small subgroup of affected children and youths, gaps in the table occurred, where the mean costs, in accordance with the relevant data protection act, were not calculated because of too small case numbers (<5). The real incurred costs for back disorders in children and youths in the 3% birthday sample were then added up. In the second step, we tried to estimate (minimum estimation) the costs incurred by back disorders in children and youths in all statutory insurants in 2002. Here, we only regarded costs of outpatient treatment and multiplied the average costs of the respective singular diagnosis by the extrapolated prevalent case numbers and finally summed up the calculated costs. Assuming that all prevalent cases created at least the average costs of one outpatient treatment, these calculations are to be regarded as a minimum estimation of the costs which are caused by dorsopathies in childhood and youth.

## Results

### Prevalence rates of diagnosis of back disorders in German children and youths

Altogether, 644,773 children and youths (female: 49.3%; male: 50.7%) were included in this evaluation. Of those, 359,922 (female: 48.9%; male: 51.1%) were between 0-14 years old and 284,851 (female: 49.7%; male: 50.3%) between 15-24 years old. Table 2 depicts the exact case number in the 3% birthday sample, the extrapolated number of all concerned children and youths of the statutory insurants as well as the respective prevalence rates (gender- and age-stratification) according to the different ICD-10 codes. It became obvious that a) the prevalence rates of diagnosis of dorsopathies range between 0.01% - 14.1%, b) many diagnosis seem to be age-dependent, especially with regard to M53 "other dorsopathies, not elsewhere classified" and M54 "dorsalgia", as well as c) that there were only rather small gender differences found in these two age-groups. In summary it can be said that M41 ("scoliosis", prevalence of diagnosis: 2.31%), M54 ("dorsalgia", prevalence of diagnosis: 1.68%), and M43 ("other deforming dorsopathies", prevalence of diagnosis: 1.33%) were found to be the main diagnoses for children (0-14 years), while M54 ("dorsalgia", prevalence of diagnosis: 12.67%), M53 ("other dorsopathies", not elsewhere classified, prevalence of diagnosis: 3.95%) and M41 ("scoliosis", prevalence of diagnosis: 3.44%) were the main diagnoses for youths (15-24 years). When extrapolating the prevalence rates to all insurants, it becomes clear that a large number of children and youths was diagnosed with back disorders and that in the year 2002 alone, about 1.4 million German children/youths were diagnosed with back pain (M54).

**Table 2:** case numbers^§ ^and prevalence rates of dorsopathies (ICD-10) in German children and youths

	age group (0-14 years)*			age group (15-24 years)**			**total collective**^**#**^		
**ICD10**	**number of cases**	**extra-polation**	**prevalence (%)**	**number of cases**	**extra-polation**	**prevalence(%)**	**number of cases**	**extra-polation**	**prevalence(%)**

M40	kyphosis and lordosis								

F	1,857	61,838	1.05	1,807	60,173	1.28	3,664	122,011	1.15

M	1,689	56,244	0.92	1,836	61,139	1.28	3,525	117,383	1.08

Σ	3,546	118,082	0.99	3,643	121,312	1.28	7,189	239,394	1.11

M41	scoliosis								

F	4,428	147,452	2.51	5,387	179,387	3.80	9,815	326,840	3.09

M	3,899	129,837	2.12	4,402	146,587	3.07	8,301	276,423	2.54

Σ	8,327	277,289	2.31	9,789	325,974	3.44	18,116	603,263	2.81

M42	spinal osteochondrosis								

F	276	9,191	0.16	984	32,767	0.70	1,260	41,958	0.40

M	319	10,623	0.17	1,525	50,783	1.07	1,844	61,405	0.56

Σ	595	19,814	0.17	2,509	83,550	0.88	3,104	103,363	0.48

M43	other deforming dorsopathies								

F	2,292	76,324	1.30	2,109	70,230	1.49	4,401	146,553	1.38

M	2,490	82,917	1.35	1,844	61,405	1.29	4,334	144,322	1.33

Σ	4,782	159,241	1.33	3,953	131,635	1.39	8,735	290,876	1.35

M45	ankylosing spondylitis								

F	10	333	0.01	84	2,797	0.06	94	3,130	0.03

M	23	766	0.01	122	4,063	0.09	145	4,829	0.04

Σ	33	1,099	0.01	206	6,860	0.07	239	7,959	0.04

M46	other inflammatory spondylopathies								

F	23	766	0.01	121	4,029	0.09	144	4,795	0.05

M	12	400	0.01	91	3,030	0.06	103	3,430	0.03

Σ	35	1,166	0.01	212	7,060	0.07	247	8,225	0.04

M47	spondylosis								

F	205	6,827	0.12	1,491	49,650	1.05	1,696	56,477	0.53

M	137	4,562	0.08	977	32,534	0.68	1,114	37,096	0.34

Σ	342	11,389	0.01	2,468	82,184	0.87	2,810	93,573	0.44

M48	other spondylopathies								

F	43	1,432	0.02	135	4,496	0.10	178	5,927	0.06

M	37	1,232	0.02	128	4,262	0.09	165	5,495	0.05

Σ	80	2,664	0.02	263	8,766	0.09	343	11,422	0.05

M49	spondylopathies in diseases classified elsewhere								

F	15	500	0.01	11	366	0.01	26	866	0.01

M	19	633	0.01	12	400	0.01	31	1,032	0.01

Σ	34	1,132	0.01	23	766	0.01	57	1,898	0.01

M50	cervical disc disorders								

F	129	4,296	0.07	527	17,549	0.37	656	21,845	0.21

M	96	3,197	0.05	256	8,525	0.18	352	11,722	0.11

Σ	222	7,393	0.06	783	26,074	0.27	1,008	33,566	0.16

M51	other intervertebral disc disorders								

F	116	3,863	0.07	1,294	43,090	0.91	1,410	46,953	0.44

M	102	3,397	0.06	1,174	39,094	0.82	1,276	42,491	0.39

Σ	218	7,259	0.06	2,468	82,184	0.87	2,686	89,444	0.42

M53	other dorsopathies, not elsewhere classified								

F	1,380	45,954	0.78	6,928	230,702	4.89	8,308	276,690	2.61

M	1,160	38,628	0.63	4,329	144,155	3.02	5,489	182,784	1.68

Σ	2,540	84,582	0.71	11,257	374,858	3.95	13,797	459,440	2.14

M54	dorsalgia								

F	3,308	110,156	1.88	19,961	664,701	14.10	23,269	774,858	7.32

M	2,730	90,909	1.49	16,116	536,663	11.30	18,846	627,572	5.76

Σ	6,038	201,065	1.68	36,077	1,201,364	12.67	42,115	1,402,430	6.53

F: female; M: male; ^§ ^exact numbers of the 3% random sample and extrapolated rounded numbers of the insured German general population; * **N (age group 0-14 years): 359,922 **(female: 176,155, male: 183,767); ** N (age group 15-24 years): 284,851 (female: 141,680, male: 143,171); ^**# **^**N (total collective, 0-24 years): 644,773 **(female: 317,835, male: 326,938); **extrapolation **to the whole German general population

### Treatment costs for back disorders in German children and youths

Table 3 shows the mean costs for in- and outpatient treatment due to singular diagnoses of back disorders. In the 3% random sample and the age group between 0-14 years, a total of 200,000 € were spent on outpatient treatment and about 209,000 € on inpatient treatment (disorders included in this cost analysis were: M41: "scoliosis", M43: "other deforming dorsopathies, M51: "other intervertebral disc disorders", and M54: "dorsalgia"). In the 3% random sample and the age group between 15-24 years about 731,000 € were spent on outpatient treatment and about 716,000 € on inpatient treatment. This age-dependent increase in costs is mainly due to the increase in case numbers and not to an increase in costs per case. The total costs for these two age groups add up to approximately €1.9 million being spent on direct treatment for back disorders (only singular diagnosis) in the 3% birthday cohort in Germany in 2002. Now, in order to make a minimum estimation of the costs incurred by all children and youths insured with statutory health insurers, we extrapolated the costs assuming that all prevalent cases received outpatient treatment at least once. Here, the overall costs would add up to approximately 100 million € for the year 2002. As several diagnoses could not be analyzed due to too small case numbers, this estimation does not depict the whole truth.

**Table 3:** number of accounting cases (n) and mean costs (per accounting case) for outpatient treatment*

	outpatient treatment because of a singular diagnosis of a back disorder					inpatient treatment because of a singular diagnosis of a back disorder				
		**age group 0-14 years**		**age group 15-24 years**			**age group 0-14 years**		**age group 15-24 years**	

**ICD10**	**gender**	**n**	**mean costs per case in € (single diagnosis)**	**n**	**mean costs per case in € (single diagnosis)**	**gender**	**n**	**mean costs per case in € (single diagnosis)**	**n**	**mean costs per case in € (single diagnosis)**

M40**	F	351	22.62	169	26.04	F	--	--	--	--
	
	M	271	21.97	260	24.42	M	--	--	--	--

M41	F	1861	24.75	1552	25.92	F	43	1,916.91	39	5,146.80
	
	M	1155	24.06	1029	25.24	M	16	5,621.80	14	1,639.58

M42**	F	55	37.80	162	31.35	F	--	--	--	--
	
	M	92	29.20	313	31.86	M	--	--	--	--

M43	F	565	22.80	412	29.14	F	11	546.24	7	4,022.69
	
	M	637	26.15	442	27.28	M	6	1,726.35	19	5,016.23

M45**	F	--	--	--	--	F	--	--	--	--
	
	M	--	--	--	--	M	--	--	--	--

M46**	F	--	--	--	--	F	--	--	--	--
	
	M	--	--	--	--	M	--	--	--	--

M47**	F	60	36.88	340	34.06	F	--	--	--	--
	
	M	38	36.42	252	36.74	M	--	--	--	--

M48**	F	6	68.87	20	37.49	F	--	--	--	--
	
	M	5	32.61	16	57.83	M	--	--	--	--

M49**	F	--	--	--	--	F	--	--	--	--
	
	M	--	--	--	--	M	--	--	--	--

M50**	F	36	49.68	161	66.58	F	--	--	--	--
	
	M	26	54.57	63	64.46	M	--	--	--	--

M51	F	31	82.24	548	90.85	F	--	--	61	2,185.05
	
	M	34	112.05	558	93.43	M	5	3,547.22	58	2,306.18

M53**	F	314	30.49	1643	31.01	F	--	--	5	401.04
	
	M	269	32.56	1075	28.37	M	--	--	5	807.64

M54**	F	866	28.04	7024	29.85	F	--	--	61	924.92
	
	M	782	27.95	6773	27.28	M	6	464.28	42	931.31

* these numbers refer to either the single diagnosis, or to cases with the relevant MSD as main diagnosis but with possibly other co-diagnoses (multiple diagnoses); ** in case of a cell entry "--", there were too small case numbers (<5) for analyzing the data without violating the data protection act; therefore mean outpatient and inpatient treatment costs for a single diagnosis could not be derived for all ICD-10 codes included in this analysis.

**ICD-10 codes: M40: **kyphosis and lordosis, **M41: **scoliosis; **M42: **spinal osteochondrosis; **M43: **other deforming dorsopathies; **M45: **ankylosing spondylitis; **M46: **other inflammatory spondylopathies; **M47: **spondylosis; **M48: **other spondylopathies; **M49: **spondylopathies in diseases classified elsewhere; **M50: **cervical disc disorders; **M51: **other intervertebral disc disorders; **M53: **other dorsopathies, not elsewhere classified; **M54: **dorsalgia

## Discussion

In the scientific literature, there seems to be large differences in health care utilization of children and youths with back pain [8,10-12]. In particular, German children with back pain were reported to seek out medical care more often (in 53% of the cases) than children and adolescents elsewhere (7-32%). Apart from back disorders which are associated with the clinical feature back pain there are other back disorders, not necessarily associated with back pain, of which we know only little with regard to prevalence rates in children and youths. This situation gave us reason to believe, that it might be necessary to reappraise the number of diagnosis of back disorders for German children and the accordingly incurred costs from a public health point of view.

In the 3% random sample of all Germans insured with statutory health insurers, which was available for scientific analysis, we found that altogether 7.4% of the insured children between 0-14 years and 25.9% of the insured youths between 15-24 years were diagnosed with a back disorder. Assuming that all of those patients experienced back pain, and considering the reported health care utilization of 53% [8], we could estimate a one year prevalence rate of back pain of about 15% in children and a one year prevalence rate of about 52% in youths. These prevalence rates seem to be very high and of course it has to be taken into account that not all back disorders are associated with back pain, e. g. scoliosis (M41). As other authors (e. g. [9]) reported a three month prevalence rate for back pain of 38.6% (in German children and adolescents aged 10 - 18 years), and one year prevalence rates for lower back pain between 7-63% in adolescents [1], our results at least seem to cover a large proportion of children and youths seeking out a doctor because of back pain.

In 2007, Kamtsiuris et al. [14] questioned German children and adolescents (and their parents) and reported that scoliosis was diagnosed in 5.2% of all examined 0-17 years olds in Germany. Girls were more often affected (6.0%) than boys (4.4%), whereby the gender difference was especially pronounced in the age groups between 11-13 years and 14-17 years. The authors themselves discuss that the reliability of the diagnosis, reported by the "patients" themselves, is to be interpreted with caution, as it cannot be differed between a suspected case and a manifest case. In contrast to these results, in our study we found slight age and gender-effects within the ICD-10 diagnosis M41 for scoliosis. This might be on the one hand due to methodological issues, as we had to use larger age groups which might have disguised slight age- and/or gender effects. On the other hand, the prevalence rates of diagnosed scoliosis seem to be lower than reported by Kamtsiuris et al. [14] which might be due to the methodological differences or due to the fact that we report the one-year prevalence rates, while Kamtsiuris et al. reported lifetime prevalence [14]. Kim et al. [15] examined scoliosis in their review in more detail and reported the prevalence of scoliosis (defined as case of scoliosis with a scoliosis curve of 10° or more) in the age group of over 10 year olds to range between 0.5-3.0%. In addition, they stated that the gender distribution is fairly even when the scoliosis-curves are small; however, there is a clear female predominance as the curve magnitude increases, with some studies quoting a ratio of 1:8 (male to female) [15]. The differences between these reports and our results are probably due to the problems which occur when using ICD-10 codes as indicator of disease, which does not allow for differentiation between scoliosis with smaller angles (<10°) vs. scoliosis with larger angles (>20°). Another point which should be kept in mind when interpreting our results is that in Germany there are standardized physical examinations for children (which the statutory health insurer pays for) and scoliosis might be identified and be coded as an incidental finding during one of these examinations.

Quite a large amount of scientific literature considers back pain in children and adolescents [e. g. [1,2,5,10,16-19]]. The one year prevalence rates in these other reports range from 3.5% - 83%, depending on localization (low back > neck > thoracic spine), age (older > younger), and gender (female > male), whereby Duggleby [20] and Olson [10] reported that only 8% and 7% of the children/youths respectively with back problems see a doctor. As described above, more recent German studies report a three month prevalence of back pain between 30.2 - 49% [7-9] and according visits to the doctor in 53% of the back pain cases [8]. Though these numbers are quite impressive, the reports tell us nothing about the diagnosis the consulted doctors made. With our analysis we can close this gap of knowledge at least in part and state, that here, altogether 1.7% of children younger than 15 years were diagnosed with back pain as compared to 12.7% of youths between 15-24 years. This noticeable increase of prevalence with age goes along with findings of other authors who reported an increasing self-reported prevalence of back pain with age in children and adolescents [e.g. [1]]. The age dependent increase might at least partially be due to the growth period in puberty and problems that arise therein. Apart from the here presented study, other literature on diagnosis of back disorders in childhood and youth is scarce. But keeping in mind the numbers of affected children and youths in Germany alone, our results uncover a need for secondary and tertiary prevention strategies for musculoskeletal disorders, especially back pain, even at an early age.

Apart from that, we found a small number of cases with the medical conditions summarized under M42 to M51 (**M42: **"spinal osteochondrosis"; **M43: **"other deforming dorsopathies"; **M45: **"ankylosing spondylitis"; **M46: **"other inflammatory spondylopathies"; **M47: **"spondylosis"; **M48: **"other spondylopathies"; **M49: **"spondylopathies in diseases classified elsewhere"; **M50: **"cervical disc disorders"; **M51: **"other intervertebral disc disorders"). This result is not surprising as degenerative diseases are hidden behind these ICD-10 codes and are not expected in large numbers in childhood and youth. The fact that we actually found cases with degenerative disorders, even at an early age, might also be due to erroneous ICD-10 coding.

To our knowledge this is the first examination that focuses on the prevalence rates and costs of back disorders in childhood and youth, which were verified by a doctor's diagnosis by means of evaluating a huge population-based data set. The use of this large data set is also our study's biggest strength [21]. Nevertheless, using the International Classification of Diagnosis (ICD-10 code) implies some methodological problems. In general, the ICD-10 codes exist of up to five-digit codes. As only three-digit codes were systematically controlled and used for statistical analysis in this large data set, we were confined to a relatively general analytical approach, leaving no room for more detailed analysis of specific diseases. However, the use of terminal codes might not have provided more specific information, as many authors reported the reliability of coding to decrease with the increasing levels of the code used (e.g. [22]).

Though the coding of medical entities with classifications is a hot topic in Germany, as the codes are used for reimbursement, the reliability of diagnosis coding based on the ICD-10 is still not clear [14]. A study of Stausberg et al. [22] found kappa-values between different coding groups on a three-digit ICD-10 level (full set of diagnosis) to range between 0.34 -0.58 (i.e. fair to moderate agreement [23]) and therefore the authors concluded, that the results concerning the reliability of diagnosis coding with ICD-10 are to be interpreted with caution. They also suggested that coding in daily practice might be worse than found in their study under "standardized" conditions. Another study reported ICD-coding to be reliable on chapter level only and found relevant coding uncertainties at three- and four-digit coding level, too (kappa-values of approximately 0.4 and 0.2 for three-digit and four-digit coding, respectively) [24]. Until now, there have been few validation studies for routine data of German statutory health insurances [25]. That is why we can only estimate the true underlying number of children with each ICD 10 diagnosis as well as the true underlying number of children with pain or activity limitations. A current study might at least allow a glimpse at the validity of coded diagnosis of the used data set with regard to the coding of General Practitioners in Berlin [26]. In this study, the authors reported a correctness of musculoskeletal disorder coding of 61% (one-digit code "M") and a correctness of M54 coding ("dorsalgia") of 71%. But while the reliability and validity of ICD-10 coding on a three-digit basis might still be under scrutiny, it is a fact that - especially with regard to population based studies in Germany - ICD-10 coding is often the only available information.

The existing data set allowed for evaluation of the mean expenditures for outpatient treatments. When looking at the 3% random sample, we found direct treatment costs of approximately € 1.9 million which shows, that the treatment costs per case are - on average - rather low. Age-effects were mainly due to increased prevalence rates and not to increases in mean costs per case. For extrapolating the costs caused by all insurants of statutory health insurers, we only considered costs accompanied by single diagnosis. Therefore, the relevant accounting case numbers were considerably smaller and do not necessarily reflect the cost profile of the "usual" case. Apart from that, we assumed that every prevalent case caused only the average direct treatment cost of outpatient treatment once per year. Inpatient treatments or outpatient treatments more than once were not included in this estimation. Therefore this analysis is only a minimum estimation of the incurring costs and gives only a first insight into the complex structure of medical accounting cases associated with back disorders. More extensive analyses are needed to provide more details. Nevertheless, the present analysis adds to the existing knowledge by the mere fact that it is based on real costs and money spent on musculoskeletal diseases in these age groups and does not rely on estimations only.

## Conclusions

Until now, information on diagnosis of back disorders in children and youths was scarce. In our study, a large population-based data set of German insurants was used to elucidate this situation. Instead of focussing on self-reported disorder or disability, this approach allows for the evaluation of medical experts' opinion and therefore gives a more objective or public health oriented insight in the topics "back pain" and "back disorders". Some of our results supported the study results of other authors, other results, like the lacking gender differences in scoliosis, were rather surprising and might be due to methodological problems which arise from using ICD-10 coding. Apart from that, the data set allowed for analysis of direct treatment costs, and disclosed that the age-dependent increasing health care costs, are due to increasing prevalence rates and not due to increasing mean costs because of more problematic cases. Unfortunately, the structure of the here examined data set, as well as other available population-based data sets is not yet cut out for addressing all open questions with regard to back disorders of children and adolescents. Standardized random validity checks of these population based data sets should be mandatory in order to use them for scientific analyses. Furthermore it might be interesting to give researchers possibilities to contribute new ideas of data linkage. Finally, an overall similar construction of large population based data sets would be very useful to conduct comparisons or to link different data sets holding different information. A closer interaction between researchers and stakeholders would be desirable, preferably on a European or even worldwide level. Nevertheless the results presented in this paper, provide insight into the prevalence rates of back disorders as well as minimum cost estimations for these disorders. These results can be used for comparison with future analyses in order to detect changes.

## Abbreviations

ICD10: international classification of diseases, version ICD-10 SGB V V1.3 (2000-2003); http://www.dimdi.de/static/de/klassi/diagnosen/icd10/ls-icdhtml-alt.htm; German statutory health insurance funds (GSHIF); **ICD-10 codes: M40: **kyphosis and lordosis, **M41: **scoliosis; **M42: **spinal osteochondrosis; **M43: **other deforming dorsopathies; **M45: **ankylosing spondylitis; **M46: **other inflammatory spondylopathies; **M47: **spondylosis; **M48: **other spondylopathies; **M49: **spondylopathies in diseases classified elsewhere; **M50: **cervical disc disorders; **M51: **other intervertebral disc disorders; **M53: **other dorsopathies, not elsewhere classified; **M54: **dorsalgia

## Competing interests

The authors declare that they have no competing interests.

## Authors' contributions

EBO and EM carried out the data analysis. EBO wrote the manuscript. CLSP, SL, TK, MMES participated in the design and coordination of the study. All authors read and approved the final manuscript.

## Pre-publication history

The pre-publication history for this paper can be accessed here:

http://www.biomedcentral.com/1471-2474/11/193/prepub
